# Daytime dysfunction may be associated with postoperative delirium in patients undergoing total hip/knee replacement: The PNDABLE study

**DOI:** 10.1002/brb3.3270

**Published:** 2023-10-04

**Authors:** Xu Lin, Ming‐Jing Pan, Xiao‐Yue Wu, Si‐Yu Liu, Fei Wang, Xin‐Hui Tang, Jia‐Han Wang, Bin Wang, Yan‐Lin Bi

**Affiliations:** ^1^ Department of Anesthesiology Qingdao Hospital, University of Health and Rehabilitation Sciences (Qingdao Municipal Hospital) Qingdao Shandong China; ^2^ Department of Gastroenterology Qingdao Hospital, University of Health and Rehabilitation Sciences (Qingdao Municipal Hospital) Qingdao Shandong China; ^3^ Department of Anesthesiology Dalian Medical University Dalian Liaoning China; ^4^ Department of Anesthesiology Weifang Medical University Weifang Shandong China; ^5^ Qingdao Clinical Medical College Nanjing Medical University Nanjing Jiangsu China

**Keywords:** biomarker, cerebrospinal fluid, daytime dysfunction, postoperative delirium, sleep characteristics, total hip/knee replacement

## Abstract

**Purpose:**

Postoperative delirium (POD) is a usual complication after total hip/knee replacement, which may be affected by sleep characteristics. However, up to now, preoperative sleep characteristics have not been evaluated as risk factors of POD. The relationship between self‐reported sleep characteristics and POD in patients has been investigated in this study.

**Patients and Methods:**

We recruited 495 cognitively intact individuals in the final analysis from the Perioperative Neurocognitive Disorder and Biomarker Lifestyle study. Sleep characteristics were tested by the Pittsburgh Sleep Quality Index (PSQI). Mini‐mental state examination was applied to assess preoperative mental status of patients. Postoperatively, we used confusion assessment method and memorial delirium assessment scale to evaluate the incidence of POD and POD severity, respectively. The cerebrospinal fluid (CSF) levels of T‐tau, P‐tau, Aβ40, and Aβ42 were detected by enzyme‐linked immune‐sorbent assay before the operation. Logistic regression, multiple linear regression, and mediation effects were performed to analyze the relationship between self‐reported sleep characteristics and POD.

**Results:**

POD was detected in 11.31% (56/495) of the patients, with logistic regression analysis showing that daytime dysfunction, P‐tau, and T‐tau were risk factors of POD, and Aβ42 was a protective factor of POD. Multiple linear regression analysis confirmed that daytime dysfunction was positively correlated with P‐tau in patients with POD. Meanwhile, compared to the patients with no postoperative delirium, the CSF levels of P‐ and T‐tau were higher in patients with POD. Furthermore, mediation analysis showed that it was probable that daytime dysfunction mediated POD through P‐tau (proportion: 12.90%) partially.

**Conclusion:**

Daytime dysfunction is a risk factor of POD preoperatively. To sum up, CSF P‐tau protein might partially mediate the influence of daytime dysfunction on POD.

**Clinical trial registration:**

This study was registered at Chinese Clinical Trial Registry (ChiCTR2000033439).

## Graphical abstract

Daytime dysfunction is one of the risk factors for POD, which may be mediated through P‐tau partially. Avoiding daytime dysfunction and maintaining a sufficient sleep might help prevent POD.

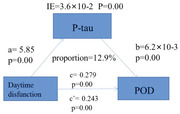

## 1 INTRODUCTION

Postoperative delirium (POD) is a common complication after surgery, which is characterized by patients’ acute changes in psychosis, including cognitive disorder, inattention, and reduced level of consciousness and possibly occurs on 1–7 days, particularly within 3 days postoperatively (Evered et al., 2018). POD is affected by various factors in perioperative period; simultaneously, the range of incidence is 4%–65% (Rudolph et al., 2011). Recently, our preliminary study had found that the incidence rate of POD was 11.4% after total hip/knee replacement (Lin etal. et al., 2020). POD could reduce the life quality and increase morbidity and mortality, meanwhile, causing a longer hospitalization and increasing the health resource costs (Shuyi et al., 2021). However, at present, there is no convincing evidence that any prophylactic measure can prevent POD; meanwhile, it is still not clear about the pathogenesis. Biomarkers such as amyloid (Aβ) and Tau protein in cerebrospinal fluid (CSF) have been confirmed to predict the POD (Bassil et al., 2020; Jia et al., 2019). Nevertheless, it was not found that independent preoperative sleep characteristics could be used as predictors of POD. Therefore, it is crucial to identify some independent preoperative sleep characteristics associated with POD, which may be predicted in the preoperative stage.

Sleep definitely helps to consolidate memories and learning. New discoveries have suggested another important role of sleep: a type of “waste management.” Neurons and glia have high metabolic rates and produce a lot of waste. The waste includes toxic substances containing lactate and two molecules (amyloid‐β (Aβ) and tau) (Anthony et al., 2021). Sleep deprivation impairs the cellular clearance of misfolded neurotoxin proteins like Aβ and tau which are involved in major neurodegenerative diseases like Alzheimer's disease (Muhammed et al., 2020). Furthermore, Aβ and tau proteins are correlated with poor self‐reported sleep quality, and the evidence supports that worse sleep quality plays a key role in early cognitive decline (Winer et al., 2021).

However, up to now, preoperative sleep characteristics as influencing factors of POD have not been evaluated. What's more, the study on the correlation between sleep characteristics and POD and their mechanisms is hard to be found.

For the above reasons, a cohort study was performed to explore the correlation between sleep characteristics and POD and mechanisms. The purpose was to find a new way to prevent the POD in the early stage. To these ends, we made the hypothesis that preoperative sleep characteristics could be influential factors of POD in patients. Afterward, we conducted the following three analyses. First of all, the correlation between sleep characteristics and POD was analyzed. And then, the correlation between T‐tau, P‐tau, Aβ40, and Aβ42 and sleep characteristics was assessed. The last is whether sleep characteristics and POD could be mediated through T‐tau, P‐tau, Aβ40, and Aβ42.

## 2 METHODS

### 2.1 Data source and extraction

Patients were made a recruitment from the Perioperative Neurocognitive Disorder and Biomarker Lifestyle (PNDABLE) study, which was assessed the risk factors and biomarkers of perioperative neurocognitive disorder (PND), in order to diagnose and prevent PND in the Han nationality patients of North China in the early stage. Ethical approval for this study (IRB number: Ethical Committee N 2020 PRO FORMA Y number 005) was provided by the Ethical Committee Qingdao Municipal Hospital, Qingdao, China (Chairman Prof Yang) on 21 May 2020. Before the patient enrollment, the study was registered at Chinese Clinical Trial Registry (Clinical registration number: ChiCTR2000033439; https://www.chictr.org.cn/showproj.aspx?proj=54473; date of registration: 1 June 2020). Then written informed consents from all subjects or legal surrogates were obtained.

### 2.2 Participants

Five hundred and twenty‐one eligible patients, among 40–90 years old, between July 2020 and December 2021 in Qingdao Municipal Hospital, scheduled to perform total hip/knee replacement, under combined spinal and epidural anesthesia, were recruited in our prospective, observational, cohort study. The inclusion criteria of the PNDABLE study include (1) 40–90 years old; (2) Han population of the northern China; (3) American Society of Anesthesiologists (ASA) grade 1 or 2; (4) good preoperative cognitive status and no language of communication confusion; and (5) educational level was sufficient to finish preoperative cognitive function and Pittsburgh Sleep Quality Index (PSQI) score [9]. The exclusion criteria include (1) major neurological diseases and central nervous system (CNS) infection, head trauma, epilepsy, and multiple sclerosis; (2) major psychological dysfunction; (3) genetic family history; (4) preoperative mini‐mental state examination (MMSE) scores less than 24; (5) serious visual and hearing impairment; and (6) unwillingness to follow the protocol or process.

### 2.3 Neuropsychological testing

Preoperatively, MMSE score was utilized to evaluate the cognitive function of the patients by a neurologist. Postoperatively, patients were followed up at 10 a.m. and 2 p.m. twice a day within 7 days (or before discharge) by an anesthesiologist. Meanwhile, POD was diagnosed according to the confusion assessment method (CAM), and POD severity was accessed according to the memorial delirium assessment scale (MDAS) (Inouye et al., 1990; Schuurmans et al., 2003). The above evaluations performed by a neurologist and an anesthesiologist did not take part in perioperative period management of the patients. The following four clinical criteria: (1) acute onset and fluctuation process; (2) inattention; (3) disorganized thinking; and (4) change of consciousness level, which meets the standards 1, 2, and 3 or 4 at the same time, could be diagnosed as POD. The CAM and MDAS that had good validity and reliability have been demonstrated in Chinese studies (Leung et al., 2008; Shi et al., 2014). Therefore, CAM and MDAS positive scores on patients postoperatively on 1–7 days (or before discharge) were recorded.

### 2.4 Self‐reported sleep characteristics testing

PSQI score was applied to test the sleep characteristics of the patients by a neurologist the day before the operation. PSQI is a self‐reported questionnaire evaluating sleep quality during the last month, including sleep quality, sleep efficiency (sleep duration in ratio to the total time spent in bed), sleep latency (minutes spent before falling asleep), sleep disturbances, nocturnal sleep duration (hours), daytime dysfunction, and sleep medication use. The total score ranges from 0 to 21, which means that the higher the score, the worse the sleep. The questionnaire also involved some special questions, for instance, “During the past month, how often have you had trouble staying awake while driving, having meals, or engaging in social activity?”, “During the past month, how often have you taken medicine (prescribed or ‘over the counter’) to help you sleep?”, and so on (Buysse et al., 1989). The total score ranges from 0 to 21, which suggests that the higher the total score, the worse the sleep.

### 2.5 Anesthesia and surgery

To avoid the influence of diverse surgical standards, all eligible patients with the same surgery department accepted total hip/knee replacement. Electrocardiography, oxygen saturation, pulse oximetry, and noninvasive blood pressure, which had fixed intervals of 3 min recording, were continuously supervised under combined spinal and epidural anesthesia. However, midazolam, nonsteroidal analgesics, dexmedetomidine, and glucocorticoid drugs were not performed during anesthesia.

Numerical rating scale (NRS) score of 0–10 (lower score showing lower pain level) (Chung et al., 2016) was applied to evaluate the pain postoperatively. Patient‐controlled intravenous analgesia, which containing 5 mg tropisetron and 2.5 μg kg^−1^ sufentanil was used for analgesia within 48 h. For postoperative analgesia, we would record if patients need to be given non‐opioid drugs.

### 2.6 Sample collection

The CSF (2 mL) was used by a polypropylene centrifugal tube to collect during combined spinal and epidural anesthesia before anesthesia. Immediately, at room temperature, it was centrifuged at 2000*g* for 10 min (Bakr et al., 2017; Pérez‐Ruiz et al., 2018), afterward, stored at −80°C, and further analysis would be performed.

### 2.7 ELISA

CSF samples were processed immediately within 2 h after standard lumbar puncture. Each sample was centrifuged at 2000 × *g* for 10 min, and CSF samples were separated and stored in an enzyme‐free EP (Eppendorf) tube (AXYGEN; PCR‐02‐C) at −80°C under the international BIOMARKAPD project for further use in the subsequent steps of this study. The optical density value of each hole was detected at the wavelength of 450 m with an enzyme marker (PerkinElmer, EnSpire) (Bakr et al., 2017; Pérez‐Ruiz et al., 2018). CSF biomarkers of POD measurements were done with other enzyme‐linked immune‐sorbent assay (ELISA) kits [Aβ42 (Fujirebio, Ghent, Belgium Lot: No. 81583), Aβ40 (Fujirebio, Ghent, Belgium Lot: No. 81585), P‐tau (Fujirebio, Ghent, Belgium Lot: 81581), and T‐tau (Fujirebio, Ghent, Belgium Lot: No. 81579)]. Moreover, the lower limits of detection of the Aβ42, Aβ40, P‐tau, and T‐tau assays in CSF were 77.38, 30.84, 11.69, and 40.65 pg/mL, respectively. All the ELISA measurements were performed by experienced technicians in strict accordance with the manufacturer's instructions, blinding to the group apportion. The samples and standards were measured in duplicates, and the means of duplicates were used for the statistical analyses. All the antibodies and plates were from a single lot to exclude variability between batches. Moreover, the within‐batch CV was <5% (4.8% for Aβ42, 3.6% for Aβ40, 4.6% for T‐tau, and 2.4% for P‐tau) and the inter‐batch CV was <15% (9% for Aβ42, 3.6% for Aβ40, 12.2% for T‐tau, and 10.9% for P‐tau).

### 2.8 Sample size

The preliminary study showed that the POD incidence rate was 12.0% and explored five covariates (daytime dysfunction, Aβ40, Aβ42, T‐tau, and P‐tau) which were included in the multivariate logistic regression; meanwhile, the loss of follow‐up rate was assumed to be 20%. Therefore, the required sample size was calculated to be 521 cases (5 × 10 ÷ 0.12 ÷ 0.8 = 521).

### 2.9 Data analysis

According to the accessed results of the POD, patients were split into two groups, POD group versus no postoperative delirium (NPOD) group. The main endpoint of this cohort study was to analyze the relationship between sleep characteristics and POD in patients. Additionally, the relationship between sleep characteristics and biomarkers in CSF and mediation analyses were also performed to analysis.

R 4.1.1 (R Project for Statistical Computing; http://www.r‐project.org) and Stata MP16.0 (Solvusoft Corporation, Inc) were used to analyze data.

The demographic, clinical data, and CSF biomarker levels were used for the comparisons between POD and NPOD groups. The *t*‐test was performed for continuous variables, whereas the chi‐square test for categorical variables. When the continuous variables were non‐normal distribution, nonparametric methods were adopted. Mann–Whitney *U* test was used to compare the difference between the two groups.

Characteristics, T‐tau, P‐tau, Aβ40, and Aβ42 were chosen as the independent variables to enter univariate logistic regression analysis. After that, the variables with *p* < .1 were established to be included in the multivariate logistic regression analysis after adjusting age, gender, weight, height, BMI, ASA, years of education, and MMSE score. And then multiple linear regression models were applied to examine the relationship between CSF biomarkers with sleep characteristics. Sensitivity analysis was carried out, including stroke (yes or no), hypertension (yes or no), and coronary heart disease (yes or no) for correction. *p* < .05 was statistically significant.

In order to assess whether T‐tau, P‐tau, Aβ40, and Aβ42 act as mediators between sleep characteristics and POD, mediation effect was performed by Stata MP16.0 (Solvusoft Corporation, Inc) following 5000 bootstrap iterations. Furthermore, the indirect effect was *p* < .05 and proportion >10%, which were set to be significant and mediating.

## 3 RESULTS

### 3.1 Patient characteristics

**FIGURE 1:**
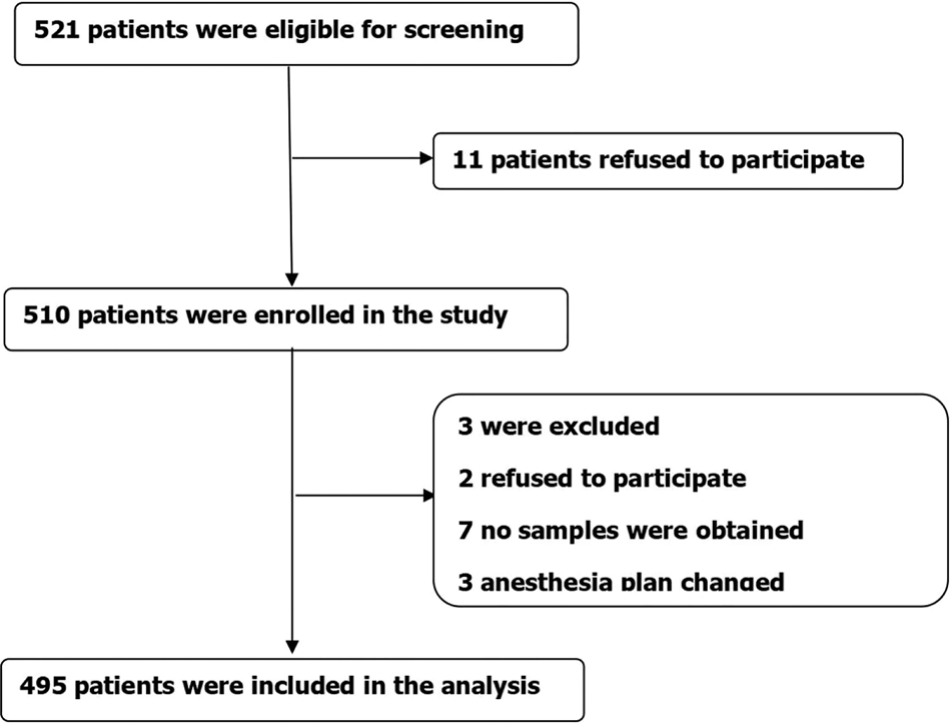
Flow diagram: The diagram shows that 521 patients were initially screened for the studies, and 495 patients were finally included in the data analysis.

We identified 521 participants who met our inclusion and exclusion, including eligible 495 participants for analysis, and 26 participants excluded. The criteria for exclusion are shown in Figure 1. The demographic and clinical data are summarized in Table 1.

**TABLE 1:** Characteristics of included participants.

	POD group (*N* = 56)	NPOD group (*N* = 439)	*p*‐Values
Gender (male/female)	30/26	226/213	.768
Age (year) (mean ± SD)	61.52 ± 9.60	60.48 ± 9.96	.497
Height (mean ± SD)	166.16 ± 7.63	166.77 ± 7.61	.720
Weight (mean ± SD)	69.06 ± 10.28	71.19 ± 11.98	.204
BMI (mean ± SD, kg/m^2^)	25.44 ± 3.55	25.45 ± 3.10	.961
Years of education, median and 25–75 percentile	9 (6,12)	9 (9,12)	.122
ASA grade, *N*(%)			.862
I	8 (14.35%)	59 (13.46%)	
II	48 (85.75%)	380 (86.64%)	
Cigarette use, yes (%)	12 (21.45%)	92 (20.95%)	.935
No (%)	44 (78.64%)	347 (79.16%)	
Hypertension, yes (%)	19 (33.94%)	140 (31.95%)	.758
No (%)	37 (66.16%)	299 (68.15%)	
Coronary heart disease, yes (%)	5 (8.94%)	33 (7.54%)	.915
No (%)*	51 (91.16%)	406 (92.56%)	
Alcohol abuse yes (%)	16 (28.63%)	126 (28.75%)	.984
No (%)*	40 (71.47%)	313 (71.35%)	
Stroke yes (%)	3 (5.46%)	9 (2.14%)	.292
No (%)	53 (94.64%)	430 (97.96%)	
Time of anesthesia (min), mean ± SD	140.38 ± 25.40	146.82 ± 23.30	.752
Time of surgery (min), mean ± SD	132.14 ± 25.34	135.76 ± 31.63	.878
Preoperative the highest MMSE score, median, and 25–75 percentile	28 (27–29)	29 (27–30)	.054
Postoperative the highest MDAS score, median, and 25–75 percentile	12 (10–14)	3 (0–5)	.000
Postoperative the highest NRS score, median, and 25–75 percentile	2 (1–2)	2 (2–3)	.083

*Notes*: The length of anesthesia was defined from the time that the anesthesiologists started the spinal anesthesia in the patients to the time when the patients were sent to the postanesthesia care unit. The length of surgery was defined from the time of initial incision to the time of the closure of the skin.

Abbreviations: ASA, American Society of Anesthesiologists; BMI, body mass index; cm, centimeter; kg, kilogram; MDAS, memorial delirium assessment scale; min, minute; mL, milliliter; MMSE, mini‐mental state examination; NPOD, no postoperative delirium; NRS, numerical rating scale; POD, postoperative delirium; SD, standard deviation.

The incidence rate of POD was detected in 11.31% (*n* = 56/495). Compared with NPOD group [29 (27–30)], there was no significant difference in preoperative MMSE score [28 (27–29)] *p* = .054] among the patients who were diagnosed with POD. Likewise, the postoperative NRS score [2 (1–2)] in POD group was not significantly different from the score [2 (2–3), *p* = . 083] in NPOD group. In addition, POD and its severity were diagnosed by CAM and MDAS scores on days 1 and 2 postoperatively in this study, which is concordant with previous study (Lin et al., 2020). Moreover, MDAS score [3 (0–5)] in NPOD group was significantly different from the patients who were diagnosed as POD [12 (10–14), *p* < .001].

### 3.2 Comparison of CSF biomarker levels between two groups

**FIGURE 2:**
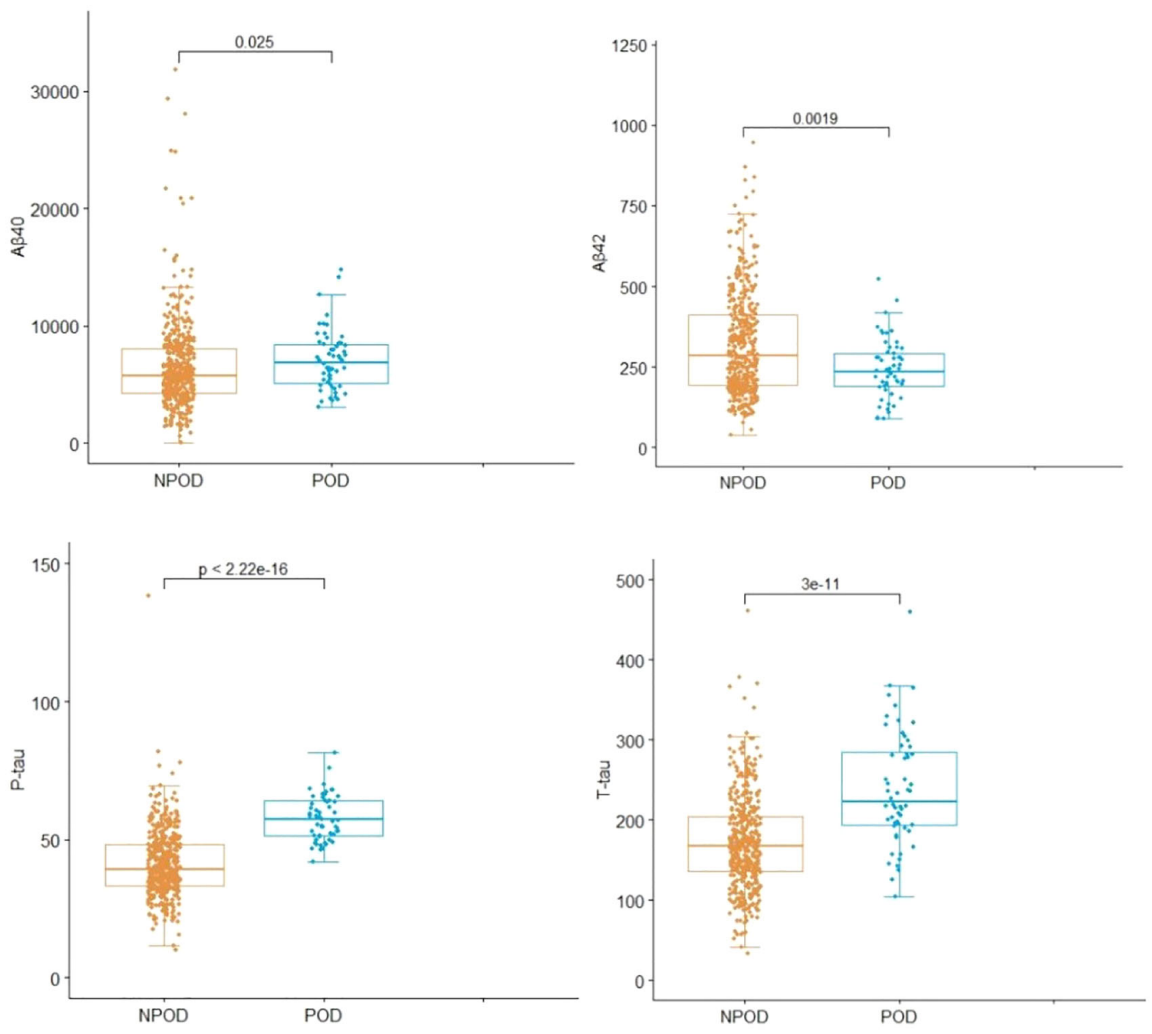
Compared with no postoperative delirium (NPOD) group, the differences in cerebrospinal fluid (CSF) levels of Aβ40, β42, T‐tau of postoperative delirium (POD) group were statistically significant (*p* < .05).

Compared with NPOD group, the differences in CSF levels of Aβ40, Aβ42, T‐tau, and P‐tau of POD group were statistically significant (*p* < .05), as shown in Figure 2.

### 3.3 Logistic regression analysis of the influencing factors of POD

In our study, after adding age, gender, weight, height, BMI, ASA, years of education, MMSE score to adjust for daytime dysfunction, Aβ42, Aβ40, P‐tau, and T‐tau, multivariate logistic regression analysis showed that daytime dysfunction (OR 13.23, CI 6.02–29.06, *p* < .001) and the increased CSF level of P‐tau (OR 1.11, CI 1.05–1.14, *p* < .001) and T‐tau (OR 1.02, CI 1.01–1.03, *p* < .001) were risk factors for POD; however, the increased CSF level of Aβ42 (OR .99, CI .99–1.00, *p* = .001) was a protective factor for POD, as shown in Table 2. Sensitivity analysis was performed by including stroke (yes or no), hypertension (yes or no), and coronary heart disease (yes or no) for correction, which was still statistically significant.

**TABLE 2:** Logistic regression analysis.

Factors of interest	Unadjusted			Adjusted		
	OR	95% CI	*p* Value	OR	95% CI	*p* Value
Daytime dysfunction	7.19	.76–68.25	*p* = .08	13.23	6.02–29.06	*p* < .001
Aβ40 (pg/mL)	1.00	1.00–1.00	*p* > .05	1.00	1.00–1.00	*p* > .05
Aβ42 (pg/mL)	.99	.99–1.00	*p* < .05	.99	.99–1.00	*p* = .001
P‐tau (pg/mL)	1.11	1.06–1.16	*p* < .001	1.11	1.05–1.14	*p* < .001
T‐tau (pg/mL)	1.02	1.01–1.03	*p* < .001	1.02	1.01–1.03	*p* < .001

*Note*: After adding age, gender, weight, height, BMI, ASA, years of education, and MMSE score, multivariate regression analysis was performed.

Abbreviations: ASA, American Society of Anesthesiologists; BMI, body mass index; CI, confidence interval; MMSE, mini‐mental state examination; OR, relative risk.

### 3.4 Relationship between daytime dysfunction and biomarkers in CSF by multiple linear regression in two groups

**FIGURE 3:**
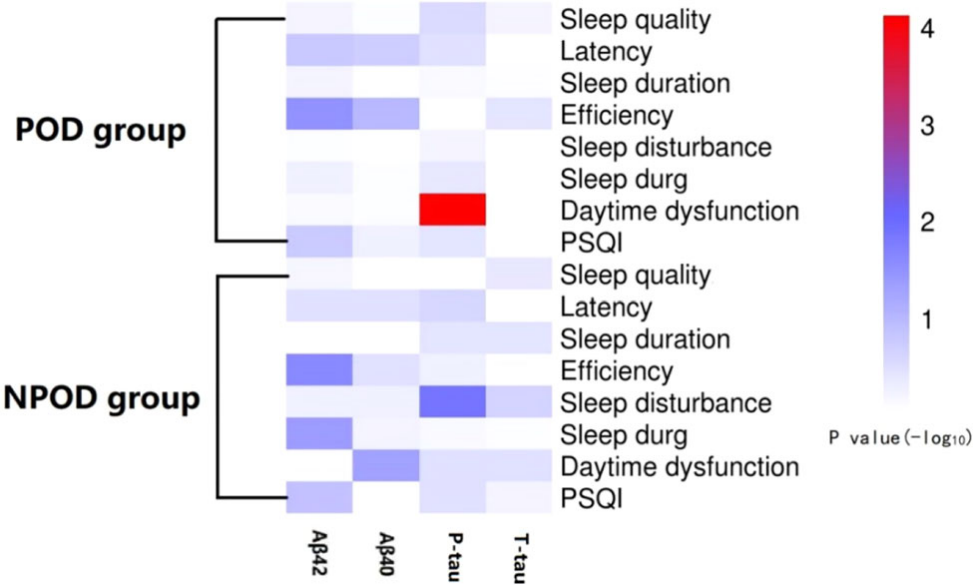
Relationship between daytime dysfunction and biomarkers in CS by multiple linear regression in two groups.

Multiple linear regression analysis showed a significantly positive association between daytime dysfunction and CSF P‐tau (*β* = .55, *p* < .001) in POD group; however, no such relationship was found in NPOD group (*p* > .05). Meanwhile, the same relationship was not found for the other three CSF biomarkers (Aβ40, Aβ42, and T‐tau *p* > .05). Relevant results of the linear regression are shown in Figure 3, Tables 3 and 4. Sensitivity analysis was performed by including stroke (yes or no), hypertension (yes or no), and coronary heart disease (yes or no) for correction, which was still statistically significant.

**TABLE 3:** Linear regression analysis in postoperative delirium (POD) group.

Daytime dysfunction	Unadjusted			Adjusted		
	*β*	95% CI	*p* Value	*β*	95% CI	*p* Value
Aβ42	.05	−30.43‐42.70	*p* > .05	.01	−39.10–40.68	*p* > .05
P‐tau	.50	3.19–8.80	*p* < .001	.55	3.40–9.59	*p* < .001
T‐tau	.03	−25.34–31.46	*p* > .05	.05	−24.96–34.97	*p* > .05

*Note*: After adding age, gender, weight, height, BMI, ASA, years of education, MMSE score, multiple linear regression analysis was performed.

Abbreviations: ASA, American Society of Anesthesiologists; BMI, body mass index; CI, confidence interval; MMSE, mini‐mental state examination.

**TABLE 4:** Linear regression analysis in no postoperative delirium (NPOD) group.

Daytime dysfunction	Unadjusted			Adjusted		
	*β*	95% CI	*p* Value	*β*	95% CI	*p* Value
Aβ42	.01	−25.82–30.63	*p* > .05	.01	−24.48–33.15	*p* > .05
P‐tau	.05	−.97–3.38	*p* > .05	.06	−0.92–3.44	*p* > .05
T‐tau	−.05	−15.64–5.28	*p* > .05	−.05	−15.86–5.68	*p* > .05

*Note*: After adding age, gender, weight, height, BMI, ASA, years of education, MMSE score, multiple linear regression analysis was performed.

Abbreviations: ASA, American Society of Anesthesiologists; BMI, body mass index; CI, confidence interval; MMSE, mini‐mental state examination.

### 3.5 Mediation analyses

**FIGURE 4:**
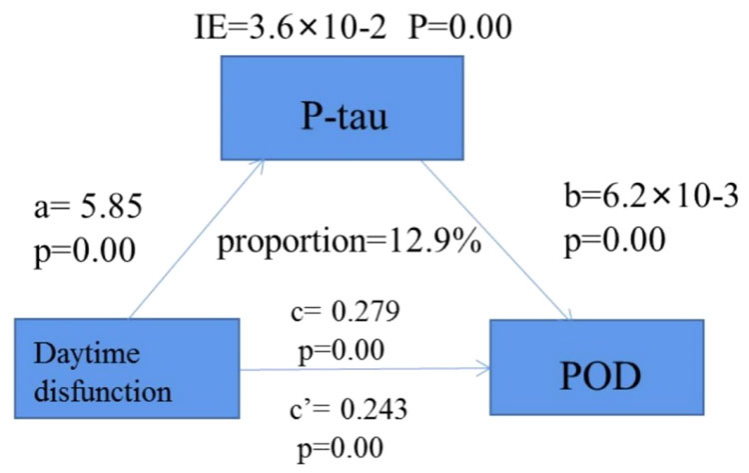
Mediation analyses with 5000 bootstrapped iterations were used to examine mediation effects of P‐tau on postoperative delirium (POD). IE, indirect effect; P‐tau, phosphorylated.

Mediation analyses showed that daytime dysfunction was likely to cause POD through P‐tau (proportion: 12.90%, *p* < .05). Relevant results are shown in Figure 4.

## 4 DISCUSSION

In this prospective, observational cohort study, we have found that daytime dysfunction is a risk factor of POD preoperatively. To sum up, CSF P‐tau protein might partially mediate the influence of daytime dysfunction on POD.

POD, an acute central nervous dysfunction, was characterized by cognitive function, consciousness, and fluctuating disturbances of concentration (Roy et al., 2019). The incidence rate of POD in this study was detected in 11.31%, which was mostly consistent with our previous study (Lin et al., 2020). Various pathogenesis is involved in POD, including inflammatory response theory, cholinergic nerve theory, Aβprotein abnormal deposition theory, and Tau protein hyperphosphorylation theory. At the same time, the concentration of biomarkers including Aβ42, Aβ40, T‐tau, and P‐tau in patients had been confirmed to be used as predictive factors for POD (McKhann et al., 2011). What's more, in the CNS, the pathophysiological changes could be better reflected with CSF, which contacted with the brain directly and was considered one of the best sources of these biomarkers (Olsson et al., 2016). Therefore, our study confirmed that the CSF levels of Aβ40, Aβ42, T‐tau, and P‐tau in POD group were significantly different from those in NPOD group. In addition, the increased concentrations of P‐tau and T‐tau were risk factors for POD; on the contrary, Aβ42 was a protective factor for POD, which is consistent with the previous research (Jia et al., 2019).

It is well known that most people spend one third of their lives being asleep. Our mood and affect, as well as our ability to attend, focus, and problem‐solving, are all directly linked to how well we sleep. Good sleepers live longer, have a reduced incidence of psychiatric disorders, and remain cognitively intact longer (Mander et al., 2013; Spiegelhalder et al., 2013; Spira et al., 2014; Lucy et al., 2015). Sleeping may rest the brain and body. Nevertheless, most organs continue to work during sleep. In particular, the brain is highly active during sleep (Nedergaard et al., 2020). The brain has a variety of ways of eliminating waste products, including local proteolytic degradation, phagocytosis by microglial cells, and passage across a porous blood–brain barrier into the circulation. Yet, a better waste management system was the glymphatic system—first suggested in the 1980s and identified definitively by Iliff et al. (2012), which is a drainage system that mingles “fresh” CSF with waste product‐rich brain interstitial fluid and flushes the fluid and waste products out of the brain and into the systemic circulation (Rennels et al., 1985). More importantly, the glymphatic system flushes out the small, soluble forms of Aβ and tau—two key features of POD, which may be neurotoxic (Holth et al., 2019). Therefore, we supposed that POD might be affected by sleep characteristics.

In our study, sleep characteristics were tested by the PSQI, a 19‐item questionnaire with high validity and reliability in retrospective self‐assessment of disturbed sleep quality over the last month, including the ensuing daytime dysfunction (Carpenter, etal., 1998). The daytime dysfunction component is based on two questions: “During the past month, how often have you taken medicine (prescribed or ‘over the counter’) to help you sleep?” and “During the past month, how much of a problem has it been for you to keep up enough enthusiasm to get things done?” (Buysse et al., 1989). Recent evidence suggests daytime sleep‐related dysfunction, which is common in cognitive impairment (Bonanni et al., 2005; You et al., 2019). Our finding was that daytime dysfunction is one of the risk factors for POD, which agreed with that participants with daytime dysfunction were more prone to cognitive impairment (Jeremy et al., 2021). Therefore, worse sleeping that results from daytime dysfunction and an insufficient sleep might lead to POD. Furthermore, it was likely to cause POD through P‐tau by mediation analyses, and the possible reason was that self‐report of poor sleep was associated with grater related pathology (such as P‐tau) in cognitively healthy adults at risk for cognitive impairment (Sprecher et al., 2021). Moreover, the present meta‐analysis demonstrated that both circulating T‐tau and P‐tau levels were significantly increased in obstructive sleep apnea (OSA) subjects when compared with non‐OSA subjects (Huang et al., 2023). However, the relationship between sleep‐related impairments and cognitive impairment pathology appeared to be bidirectional (Ju et al., 2013), which required further research. Therefore, sleep health from daytime sleep‐related function may be a tractable target for early intervention to attenuate cognitive impairment pathology and reduce incidence rate of POD.

The advantages of our PNDABLE study are including a large sample size, which bases on cognitive intact individuals preoperatively. Most importantly, we first studied and analyzed the relationship between preoperative self‐reported sleep characteristics and POD, as well as sleep characteristics and POD biomarkers.

Nonetheless, our study also has some limitations. First, based on prospective, observational, cohort studies, the results of the self‐reported sleep questionnaire could not provide a necessary causal relationship. Therefore, the effect of management intervention on preoperative patients needs to be verified by future studies, which is also our next research direction. Second, the PSQI used here only reflects the sleep characteristics of short period, rather than long period, which depends on further research. Third, this study was a single‐center study, which could be further validated by multicenter study in the future.

## 5 CONCLUSIONS

Daytime dysfunction is a risk factor of POD preoperatively. To sum up, CSF P‐tau protein might partially mediate the influence of daytime dysfunction on POD. Avoiding worse sleeping which results from daytime dysfunction and maintaining a sufficient sleep may help prevent POD.

## AUTHOR CONTRIBUTIONS

XL contributed to study design, data collection, statistical analysis, and manuscript preparation. YXW and YSL performed ELISA. JMP involved in data collection. FW, XHT and JHW performed neuropsychological testing. YLB and BW contributed to study concept and design, manuscript preparation and review. XL and YXW performed statistical analysis.

## CONFLICT OF INTEREST STATEMENT

The authors declare that they have no known conflicts of interest or personal relationships that could have appeared to influence the work reported in this paper.

### PEER REVIEW

The peer review history for this article is available at https://publons.com/publon/10.1002/brb3.3270.

## Data Availability

The data that support the findings of this study are available from the corresponding author upon reasonable request.
